# Community-Based Wellness and Prevention Programs: The Role of Medicare

**DOI:** 10.3389/fpubh.2014.00189

**Published:** 2015-04-27

**Authors:** Erin Murphy Colligan, Naomi Tomoyasu, Benjamin Howell

**Affiliations:** ^1^Centers for Medicare and Medicare Services, Baltimore, MD, USA; ^2^CVS Health, Woonsocket, RI, USA

**Keywords:** Medicare, wellness, community-based programs, Affordable Care Act, aging, fitness, falls prevention, self-management

Community-based wellness and prevention programs have long served to address the needs of an aging population with multiple chronic diseases. Title IIID of the Older Americans Act, passed in 1987, called for the Administration on Aging (AoA) to fund “education and implementation activities that support healthy lifestyles and promote healthy behaviors (1).” AoA, now a part of the Administration for Community Living (ACL), has continued to review the evidence base for wellness and prevention programs and launched the Evidence-Based Prevention Program in 2003 to increase access to such programs for older adults (1, 2). The National Council on Aging, in conjunction with AoA, operates a clearing house of evidence supporting wellness interventions and provides technical assistance to organizations implementing the interventions (3).

Unfortunately, community-based wellness and prevention programs have yet to be incorporated into the continuum of care for Medicare recipients. At the Centers for Medicare and Medicaid Innovation (CMMI), we have embraced a broader view of addressing prevention and wellness in our beneficiaries. We now have several efforts underway to bridge the gap between clinical and community-based care. In this article, we discuss the results of research to date, describe current efforts to evaluate and engage with community-based wellness and prevention programs, and outline some challenges that we have recognized in fully integrating these interventions into the Medicare system.

Recognizing the potential of community-based wellness and prevention programs to improve health and reduce medical costs among Medicare beneficiaries, Congress called upon the Secretary of Health and Human Services to evaluate these programs under Section 4202(b) of the Affordable Care Act. To address this statute, CMMI first directed an evidence review and environmental scan of existing wellness programs, which Altarum Institute completed in 2011. Altarum rated the strength of peer-reviewed literature surrounding a variety of wellness and prevention programs and found several with the potential to benefit older adults (4). The results of this first phase of research informed the selection of promising programs for the second phase of research, a retrospective analysis of claims-based outcomes for Medicare recipients who participated in select wellness interventions from 1999 to 2012. Acumen LLC completed the retrospective study in January 2013, and their findings along with those of Altarum formed the basis for a Report to Congress delivered In November 2013 (5).

As described in the Report to Congress, Acumen found statistically significant total medical cost savings for four established wellness programs: EnhanceFitness (EF), Arthritis Foundation Exercise Program (AFEP), Arthritis Foundation Tai Chi Program (AFTCP), and Matter of Balance (MOB). Two additional programs, the widely disseminated Chronic Disease Self-Management Program (CDSMP) and the Arthritis Foundation Aquatic Program (AFAP), demonstrated reductions in inpatient hospital costs, which indicate a potential for future long-term savings. One common element of the programs that were associated with total cost savings is that they all included consistent physical activity to prevent and manage chronic conditions. These findings suggest that physical fitness may be a critical mechanism through which to achieve benefits in health, utilization, and cost outcomes.

The next phase of research under the 4202(b) legislation is currently underway: a prospective evaluation of new participants in wellness programs. Acumen LLC, in partnership with Westat, is conducting this study, with initial results expected in 2016. While the retrospective study showed some promising results, it was limited by the inherent selection bias in beneficiaries who voluntarily enrolled in wellness programs. Although Acumen matched program participants to non-participants based on clinical and sociodemographic factors as well as cost and utilization patterns, there still may be an unobservable difference in people who seek out community-based wellness and prevention programs. The prospective study will attempt to address selection bias through a population survey to measure Medicare beneficiaries’ readiness to engage in wellness interventions, which will allow for more precise matching between participants and controls based on personal motivation factors. The survey will also gauge knowledge of and interest in wellness programs, which will inform efforts to scale interventions.

Along with the 4202(b) evaluation, CMMI is considering proposing an innovative new community-based service delivery model, the Accountable Health Community (AHC), aimed at achieving better care and lower health care costs for beneficiaries with highly prevalent chronic diseases within a defined geographic area. AHCs would utilize funds from CMMI as well as from other public and private funding sources to provide a full range of preventive, non-medical, and community-based health services. CMMI funds would be used to strengthen the local infrastructure and provide the “glue” to coordinate and align services provided by clinical and social service partners as well as private payers within communities. The model is designed to create a foundation upon which these integrated community services are available and leveraged to achieve the greatest impact on CMS beneficiaries.

These efforts demonstrate a commitment on the part of CMMI to incorporate community-based wellness and prevention programs in the continuum of care for Medicare beneficiaries. Nonetheless, there are challenges that remain in terms of fully integrating community-based interventions into the Medicare payment system. First, the cost/benefit ratio of implementation costs to Medicare savings needs to be made clear before wellness programs are widely offered to Medicare beneficiaries. Current programs are primarily funded through grants and do not take into consideration the costs of delivering the interventions on a larger scale and whether or not the payer would receive returns on investment. The prospective study will try to address this issue by calculating the cost to administer programs in light of savings accrued to Medicare. Furthermore, community-based wellness and prevention programs rely on a non-clinical workforce of lay instructors that do not fit into current Medicare payment structures. Thus creating a benefit for community-based prevention and wellness intervention will require reconsideration of how CMS can compensate non-traditional health providers. Medicaid recently allowed for a broader range of providers to administer preventive services to “(1) prevent disease, disability, and other health conditions or their progression; (2) prolong life; and (3) promote physical and mental health and efficiency,” as long as the services are recommended by a physician or licensed provider (6). Medicare, however, has yet to incorporate non-clinical providers into the reimbursement system. We hope that our current projects will offer more perspective into these challenges and provide insights on how to make community-based wellness and prevention programs accessible and available to a broader population of Medicare beneficiaries.

## Conflict of Interest Statement

The authors declare that the research was conducted in the absence of any commercial or financial relationships that could be construed as a potential conflict of interest.

This paper is included in the Research Topic, “Evidence-Based Programming for Older Adults.” This Research Topic received partial funding from multiple government and private organizations/agencies; however, the views, findings, and conclusions in these articles are those of the authors and do not necessarily represent the official position of these organizations/agencies. All papers published in the Research Topic received peer review from members of the Frontiers in Public Health (Public Health Education and Promotion section) panel of Review Editors. Because this Research Topic represents work closely associated with a nationwide evidence-based movement in the US, many of the authors and/or Review Editors may have worked together previously in some fashion. Review Editors were purposively selected based on their expertise with evaluation and/or evidence-based programming for older adults. Review Editors were independent of named authors on any given article published in this volume.
